# The influences of Taiwan's National Health Insurance on women's choice of prenatal care facility: Investigation of differences between rural and non-rural areas

**DOI:** 10.1186/1472-6963-8-67

**Published:** 2008-03-29

**Authors:** Likwang Chen, Chi-Liang Chen, Wei-Chih Yang

**Affiliations:** 1 Centre for Health Policy Research and Development, National Health Research Institutes, No.35 Keyan Road, Zhunan Town, Miaoli County 350, Taiwan; 2 Institute of Public Health & Department of Social Medicine, School of Medicine, National Yang-Ming University, Taipei City 112, Taiwan; 3 The Department of Accounting, The College of Business, Chung Yuan Christian University, Chung-Li City, Taoyuan County 320, Taiwan; 4 Department of Accounting, College of Management, National Taiwan University, Taipei City 106, Taiwan

## Abstract

**Background:**

Taiwan's National Health Insurance (NHI), implemented in 1995, substantially increased the number of health care facilities that can deliver free prenatal care. Because of the increase in such facilities, it is usually assumed that women would have more choices regarding prenatal care facilities and thus experience reduction in travel cost. Nevertheless, there has been no research exploring these issues in the literature. This study compares how Taiwan's NHI program may have influenced choice of prenatal care facility and perception regarding convenience in transportation for obtaining such care for women in rural and non-rural areas in Taiwan.

**Methods:**

Based on data collected by a national survey conducted by Taiwan's National Health Research Institutes (NHRI) in 2000, we tried to compare how women chose prenatal care facility before and after Taiwan's National Health Insurance program was implemented. Basing our analysis on how women answered questionnaire items regarding "the type of major health care facility used and convenience of transportation to and from prenatal care facility," we investigated whether there were disparities in how women in rural and non-rural areas chose prenatal care facilities and felt about the transportation, and whether the NHI had different influences for the two groups of women.

**Results:**

After NHI, women in rural areas were more likely than before to choose large hospitals for prenatal care services. For women in rural areas, the relative probability of choosing large hospitals to choosing non-hospital settings in 1998–1999 was about 6.54 times of that in 1990–1992. In contrast, no such change was found in women in non-rural areas. For a woman in a non-rural area, she was significantly more likely to perceive the transportation to and from prenatal care facilities to be very convenient between 1998 and 1999 than in the period between 1990 and 1992. No such improvement was found for women in rural areas.

**Conclusion:**

We concluded that women in rural areas were more likely to seek prenatal care in large hospitals, but were not more likely to perceive very convenient transportation to and from prenatal care facilities in the late 1990s than in the early 1990s. In contrast, women in non-rural areas did not have a stronger tendency to seek prenatal care in large hospitals in the late 1990s than in earlier periods. In addition, they did perceive an improvement in transportation for acquiring prenatal care in the late 1990s. More efforts should be made to reduce these disparities.

## Background

Although there is some dispute over how prenatal care can improve child health, its potential in this regard is well recognized [1-3]. It is thought to benefit child health and reduce maternal mortality, and it is thought to be a good vehicle for the delivery of health care, health education, and psychosocial services to women [4,5]. Ensuring access to adequate prenatal care has been an important task for both developed and developing countries; thus, barriers to the utilization of prenatal care services have attracted much attention from health policy researchers.

Level of utilization of prenatal care has been associated with age, marital status, educational level, occupation, income, higher parity, difficulties in dealing with health service organizations, and health insurance status [6-10]. It has also been associated with conditions during pregnancy, including gaining excess weight during pregnancy, having a baby for the first time, carrying twins or triplets, being at a higher obstetric risk, being attended to by a doctor rather than other types of caregivers, and switching to another health care facility during pregnancy [6,10]. Among various factors associated with prenatal care utilization, the provision of public health insurance to cover prenatal care has been a policy instrument of interest to many advanced or newly industrialized countries.

Literature specifically discussing the effect of public health insurance on prenatal care utilization is quite limited. Some studies have found that it has a positive effect. Griffin et al. (1999), comparing enrollees of a Medicaid managed care program in Rhode Island and their counterparts with private insurance, concluded that the implementation of the Medicaid program resulted in significant improvement in adequacy of prenatal care utilization [11]. Chen et al. (2001), using data from two cross-sectional surveys conducted in 1989 and in 1996 to investigate the utilization pattern of prenatal care in Taiwan, found that utilization of expensive prenatal services, such as amniocentesis and German measles testing, was substantially higher in 1996 than in the late 1980s, suggesting that the implementation of the NHI may induce greater demand for expensive prenatal services [12]. In contrast, they also found that from late 1989 to 1996 the proportion of women receiving consultation services remained stable and the proportion of women receiving family planning consultation declined [12].

Results from another study using data from the same Taiwanese cross-sectional surveys showed that Taiwanese women were more likely to receive adequate prenatal care in 1996, one year after the launch of the NHI, than in 1989 [6]. That same study found that before the NHI was inaugurated, female farmers and blue-collar workers used prenatal care services more frequently than women in other occupations, but afterwards female civil servants were the group seeking prenatal care most frequently [6]. It has also been found that Taiwanese women who visited clinics were more likely to receive adequate prenatal care than those visiting hospitals after the NHI started, though this was not found in the late 1980s [6,10]. However, previous studies have not well investigated the differences in the choice of prenatal care facility and perceived convenience in transportation for acquiring such care between women living in areas with different levels of urbanization in Taiwan.

Since it established its national health insurance program in 1995, Taiwan has stopped using its public health system of community health care centres as the major vehicle of providing free prenatal care, and began to provide it through the NHI system, which allows expectant mothers to seek prenatal care from a variety of healthcare facilities. Before 1995, Taiwan already had three major types of public insurance programs covering about 57% of the population. The Labor Insurance program, launched in 1950, covered employees such as workers of government-run enterprises, private company employees, blue-collar employees and members of professional unions who were over 15 years of age and under 60 years of age. Established in 1958, the Government Employee Insurance (GEI) program, covered officers and full-time employees of government agencies, teaching and administrative staff of government-owned schools and private schools, and retirees from these organizations. In 1988, the Farmer's Insurance program extended coverage to members of farmers' associations and individual farmers who were over 15 years old. The three programs had similar benefits packages, covering outpatient visits, hospitalisation, diagnostic tests and prescription drugs. However, only the GEI program covered dependents, and offered free prenatal care to its enrolees through health care facilities that had contracts with this public health program.

While GEI was the only public health insurance program to provide prenatal care before the launch of the NHI, Taiwan's government had been promoting prenatal care long before the introduction of the NHI system through its public health system of community health care centres. Before 1980, each township had its own health station. Additionally, by recruiting physicians from non-rural areas to support services in rural public health stations, Taiwan's government had strengthened the functions of these public health stations and improved health care resources in remote areas by the mid-1990s. Taiwan's public health stations played an important role in providing health care to people in rural areas. For instance, most Taiwanese people in remote areas thought that the local public health station was the health care facility most close to them [13]. The public health stations also played an important role in disseminating health knowledge in rural areas in the early 1990s. A study in 1991 indicated that the major source of medical information for families in rural areas in Taiwan came from the public health nurses from these stations who made home visits [14]. Through this network of public health stations, Taiwan's government delivered free prenatal care to pregnant women long before the NHI was implemented.

Taiwan's NHI in 1995 substantially extended insurance coverage to all its citizens with an equal and comprehensive benefits package. By the end of 1995, it covered 97% of the population; by 1998, the coverage was over 99%. Since 1995, Taiwan's government has been providing ten free prenatal care visits to each pregnant woman in Taiwan through the NHI delivery system. Almost all hospitals and over 90% of clinics have contracts with the NHI program, and this has substantially expanded women's choice of health care facilities from which they can seek prenatal care. Because of the increase in facilities, it would seem natural that the NHI may substantially reduce the travelling costs for seeking prenatal care. Nevertheless, physician and health facility registration data in the NHI database show that the density of gynaecologists and obstetricians and that of health care facilities providing gynaecologic and the obstetric care were a lot lower in rural areas than non-rural areas, suggesting that the NHI could substantially expand women's prenatal health care choices only in non-rural areas.

There has not been much research on the effect of public health insurance on utilization patterns of prenatal care. What little there is has focused on the number of prenatal care visits made and when they are made. This study compares the differences in how women in rural and non-rural areas chose prenatal care facilities before and after the implementation of the NHI. To do this, we analysed women's responses to a national survey in Taiwan. We focused on their replies to questions about "the type of major health care facility used," and "the convenience of transportation to and from prenatal care facility." We compared the difference in how they answered these questions for children born before and after the implementation of the NHI.

The first indicator was chosen because it can be related to quality of care. Although many people tend to believe that health care delivered in large hospitals, which are usually better equipped, is better than that provided by clinics, some studies in Taiwan have suggested that one would be more likely to get more adequate prenatal care in a clinic than in a large hospital [6,10]. The second indicator, "the convenience of transportation to and from prenatal care facility," can be related to "the physical accessibility" to care. This indicator is worthy of investigation, especially for Taiwan, where one of the major goals of its national health insurance program was to improve accessibility to health care.

Results obtained by this investigation may help set future directions of prenatal care provision in Taiwan, and may also be used by countries hoping to improve prenatal care delivery. In particular, Taiwan's case offers a unique opportunity to study the influences of national health insurance on women's choice of prenatal care facility in a situation in which they were once able to receive it free at designated public health facilities but now can seek it in any facility they want to go to as long as it has a contractual agreement with the Bureau of National Health Insurance. Other countries may also face such policy options in the future. Therefore, Taiwan's lesson can be useful to them.

## Methods

### Study design and data

We wanted to know whether Taiwan's National Health Insurance influenced the aforementioned indicators differently in rural and non-rural areas. We examined what disparities existed in these indicators between women in rural and non-rural areas in various periods before and after the NHI was launched. Specifically, we selected two periods before NHI, which were the early 1990 to 1992 and 1993 to the early 1995, and two periods after NHI, which were 1996 to 1997, and 1998 to 1999.

This study used second-hand data. The data were collected retrospectively from a national face-to-face interview survey conducted by Taiwan's National Health Research Institutes in the latter half of 2000. This national survey, which was for investigating child health and related health care utilization, collected data for two national representative samples – one for children born between March 1 of 1995 and February 28 of 1996 (1,853 children), and the other for children born between March 1 of 1996 and February 28 of 1999 (2,207 children). The survey was administered to the caregivers of these children. The respondents were also asked to report information for these children's siblings born on or after March 1 of 1990. Therefore, the database of this survey included records for children born between 1990 and 2000. The response rate was 76%, with over 98% of the respondents being the children's mothers. In total, the survey collected data for 7,817 children in 3,934 families.

For each child, the survey data include the child's and the mother's demographic background, the socioeconomic conditions of the family, the child's location of residence, and childcare and health care utilization for the child. Regarding the choice of prenatal care facility, the mother was asked to reply the following question: "What was the name of your major health care facility for acquiring prenatal care during this pregnancy, and in which township was this facility located?" Based on information collected by this question, the interviewer subsequently recorded the type of the health care facility referring to a corresponding database constructed by Taiwan's Department of Health. The mother was further asked to evaluate her perceived convenience of transportation to and from this health care facility, by the following question: "Was it convenient for you to go to this prenatal care facility?" The corresponding responses were "very inconvenient," "somewhat inconvenient," "moderate," "convenient," and "very convenient."

We extracted data from this database for our study based on the following criteria. First, we excluded records reported by caregivers other than the mothers. Second, we excluded records for children born between March 1 of 1995 and December 31 of 1995, because the mothers would be pregnant both before and after the NHI was implemented. Third, we also excluded children born in 2000, a special year in Taiwan. It was the start of the new millennium, and also the year of dragon, the preferred year for having babies for Taiwanese, especially male babies. Because this year was not a typical year in Taiwan in terms of the level of fertility as well as the obstetrical care market for years after the mid-1990s, but there were not enough children born in 2000 to form a sub-sample, we excluded cases for 2000. Fourth, we excluded children whose main caregivers in infancy were not the mothers, since we had to use information on the district where a child lived in infancy to construct a proxy variable reflecting the mother's living place during pregnancy. Fifth, we excluded children whose mothers were covered by the GEI during pregnancy, since women with the GEI were a special group with public insurance covering prenatal care in the pre-NHI period.

We were left with 4,820 children after these selection processes. Within these cases, 1,575 were children from mothers who contributed one record each to these 4,820 cases, and the other 3,245 cases were children from mothers who contributed at least two records each to these 4,820 cases. This paper reports results based on data for the 1,575 children, out of a concern regarding correlation among multiple cases from a same woman. While our analytical sample did not end up being a nationally representative sample for children born in these years, it should still be appropriate for this present study, as the focus of this study is based on multivariate statistical analysis, and a rich set of explanatory variables were included in its estimation.

Unfortunately, we did not have a sufficiently large sample size to compare the experiences of prenatal care utilization for a specific cohort before and after the implementation of the NHI. We could only find 168 women who gave a birth between March 1 of 1990 and February 28 of 1995 and another in the period between 1998 and 1999. This prohibited us from comparing a cohort of women's behaviours before the NHI and in the late 1990s.

### Sample characteristics

The characteristics for the 1,575 cases are presented in Table 1. As shown in Table 1, in our sample, women who delivered children during the pre-NHI period tended to deliver their first children, and also reported a younger age at delivery. This is related to the data collection process for the NHRI survey, from which we obtained our second-hand research data. To be included in the original sample for this survey, a child born in the pre-NHI period must have had at least one younger sibling born in the post-NHI period. Therefore, the sampled children born in the pre-NHI period were more likely to have a lower birth order than those born after the NHI was implemented, and women in the sample also tended to report a younger age at delivery for children born before the launch of NHI. In our analytical sample, each child born in the pre-NHI period had a younger sibling who was excluded in our sample selection process. Most of such siblings were born between March 1, 1995 and December 31, 1995, and this is related to the reasons why the proportion of males was higher and more children were born in rural areas in the cohort of children born in 1993–1995. Rural women tend to have a shorter space between having their first two births, and appear to have more baby boys. That there were more boys in the sample in years after the mid-1990s is consistent with the facts that the traditional Taiwanese culture has a strong son preference, and that high sex ratios at birth have been observed. There has been some argument over artificial selection of the gender of offspring by medical technology since the 1990s [15,16].

**Table 1:** Sample characteristics

	Children born in 1990/3/1~1992/12/31 (n = 190) %	Children born in 1993/1/1~1995/2/28 (n = 230) %	Children born in 1996/1/1~1997/12/31 (n = 696) %	Children born in 1998/1/1~1999/12/31 (n = 459) %
*The gender of the child*				
Male	51.05	57.39	54.31	53.59
Female	48.95	42.61	45.69	46.41
*The birth order of the child*				
Rank 1	71.05	91.30	46.55	56.86
Rank 2	23.68	6.52	45.40	38.13
Rank 3 or higher	5.26	2.17	8.05	5.01
*Maternal age when the child was born*				
Younger than 30	86.84	86.09	66.67	69.93
30–34	13.16	12.61	25.00	23.31
35–39	0.00	1.30	7.47	6.10
40 or over	0.00	0.00	0.86	0.65
*Any abnormal condition during this pregnancy*				
Yes	11.58	9.13	10.20	12.20
No	88.42	90.87	89.80	87.80
*The urbanization level of residence during this pregnancy*				
Large cities	47.89	38.70	46.70	47.06
Small cities/towns	34.21	39.13	37.21	36.82
Rural areas	17.89	22.17	16.09	16.12
*The year when the mother was born*				
After 1970	10.53	31.74	43.39	60.57
1960–1969	82.11	64.78	52.44	37.91
Before 1959	7.37	3.48	4.17	1.53
*The number of children for the mother in 2000 (the survey year)*				
1 child	0.00	0.00	30.46	42.05
2 children	67.37	83.91	58.48	51.20
3 children or over	32.63	16.09	11.06	6.75
*Maternal ethnic background*				
Fu-Chien	75.79	78.70	70.55	73.64
Hakka	12.11	8.26	10.63	8.93
Mainland China	8.95	9.57	14.08	11.33
Aboriginal/foreign	3.16	3.48	4.74	6.10
*Maternal marital status in 2000*				
Currently married	98.95	99.13	96.98	98.69
Not currently married	1.05	0.87	3.02	1.31
*Maternal educational level in 2000*				
No more than junior high school	25.26	22.61	15.80	14.81
Senior high school	54.21	55.22	57.18	57.95
College or above	20.53	22.17	27.01	27.23
*Monthly family income in Taiwanese dollar in 2000*				
Less than 50,000	38.42	40.87	39.37	45.32
50,000–69,000	38.95	37.83	36.49	31.37
70,000 or more	22.63	21.30	24.14	23.31

For each cohort of children, almost all of their mothers were married in 2000. The proportion of aboriginal or foreign-born mothers was higher for children born after 1995. The proportion of mothers who immigrated to Taiwan from Mainland China was also higher for children born after 1995. This is consistent with the fact that Taiwan has more and more children who have mothers immigrating to Taiwan from Mainland China or some countries in south-eastern Asia. Women who delivered children after 1995 tended to have more education, and those delivering children in the late 1990s tended to have lower family income. This should be related to the fact that the mothers of children born after 1995 tended to be younger than the women delivering children during the pre-NHI period.

Table 2 compares the choices of prenatal care facility of women living in rural areas and those in non-rural areas. These descriptive statistics suggest that women living in rural areas were less likely to choose medical centres or regional hospitals (large hospitals) than women in non-rural areas in the pre-NHI period. However, women in rural areas were more likely to choose large hospitals as their major health facility in the late 1990s than earlier in the 1990s, while women in non-rural areas did not have such a trend. A smaller proportion of women in rural areas perceived very convenient transportation to and from prenatal care facility in the late 1990s than earlier. In contrast, more women in non-rural areas felt that their transportation for acquiring prenatal care was very convenient in years after 1992 than in 1990–1992.

**Table 2:** The type of major health facility used for prenatal care

	Rural areas				Non-rural areas			
		
	Children born in 1990~1992 (n = 34) %	Children born in 1993~1995 (n = 51) %	Children born in 1996~1997 (n = 112) %	Children born in 1998~1999 (n = 74) %	Children born in 1990~1992 (n = 156) %	Children born in 1993~1995 (n = 179) %	Children born in 1996~1997 (n = 584) %	Children born in 1998~199 (n = 385) %
Medical centres or regional hospitals as the type of major health care facility used for prenatal care	8.82	9.80	11.61	25.68	27.56	26.26	30.31	25.71

Very convenient transportation to and from prenatal care facilities	35.29	25.49	29.46	17.57	26.92	37.99	34.42	37.14

### Empirical specification and statistical models

This study defined three types of health care facilities, and four levels of the convenience of transportation. (The detailed definitions of the outcome variables can be found in Table 3.) The indicator regarding "the type of major health care facility used" is a 3-point nominal variable, and we adopted the multinomial model to investigate factors related to this indicator [17]. We used a 4-point ordinal variable as the indicator for "the convenience of transportation" and applied the ordered probit model in our multivariate analysis [17]. The Huber/White/sandwich estimator was employed to obtain robust variance estimates. This method is a commonly used estimator of standard errors, and it is robust without assuming that the standard errors are independent from the explanatory variables and are identically distributed [18]. We used the Stata Statistical Software for our multivariate analysis.

**Table 3:** Definitions of the outcome variables and major explanatory variables in multivariate analysis

Variable	Definition of the variable
Outcome variable	
The type of major health care facility used for prenatal care	1 = obstetrics in medical centres or regional hospitals; 2 = obstetrics in local hospitals; 3 = obstetric clinics, health stations, midwives or others.
The convenience of transportation to and from prenatal care facilities	1 = inconvenient (very inconvenient or somewhat inconvenient); 2 = moderate; 3 = convenient; 4 = very convenient.
Major explanatory variables	
*Rural*	= 1 if a mother lived in a rural area during pregnancy, and 0 otherwise.
1993_1995	= 1 if a pregnancy was during January 1, 1993 and February 28, 1995, and 0 otherwise.
1996_1997	= 1 if a pregnancy was during January 1, 1996 and December 31, 1997, and 0 otherwise.
1998_1999	= 1 if a pregnancy was during January 1, 1998 and December 31, 1999, and 0 otherwise.
Rural*1993_1995	The interaction term of "*Rural*" and "1993_1995"
Rural*1996_1997	The interaction term of "*Rural*" and "1996_1997"
Rural*1998_1999	The interaction term of "*Rural*" and "1998_1999"

We specified three major types of explanatory variables – the time period a child was born in, the level of urbanization in the area where a woman resided while being pregnant, and the interaction of these two factors. In addition to these major explanatory variables, we also controlled for a detailed set of other factors: some related to children, and some related to the mother and the family. The child characteristics included gender and birth parity. The maternal characteristics included her age when she bore the child, her abnormal obstetric health problems during this pregnancy, the year when she was born, and her ethnic background. Maternal characteristics also included the number of children she had, and her marital status and educational attainment at the time of the interview. Regarding the family, we included "the average monthly family income in the year in which a mother was interviewed" as an explanatory variable. Due to data availability, the explanatory variables included a mother's marital status and educational attainment at the time of interview, and the average monthly family income of the year in which the interview was administered, instead of those variables at the time of pregnancy.

The empirical specification used to compare the influences of the NHI on the choice of prenatal care facility between women living in rural areas and those in non-rural areas is as follows:

$$\begin{array}{l} Y_{i}=\beta_{0}+\beta_{1}Rural_{i}+\beta_{2}1993\_1995_{i}+\beta_{3}1996\_1997_{i}+\beta_{4}1998\_1999_{i}\\ +\beta_{5}Rural_{i}*1993\_1995_{i}+\beta_{6}Rural_{i}*1996\_1997_{i}+\beta_{7}Rural_{i}*1998\_1999_{i}\\ +\beta_{8}^{'}X_{i}^{c}+\varepsilon_{i} \end{array}$$

*Y *is one of the two indicators mentioned previously. *X*^*c *^denotes explanatory variables other than the major ones. 1993_1995, 1996_1997, and 1998_1999 were used to capture the time trend, with the base period being from March 1, 1990 to December 31, 1992. The three interaction terms were for investigating the differences in the time trend between rural women and their counterparts in non-rural areas (The detailed definitions of the major explanatory variables can be found in Table 3).

### Estimation of changes in the relative probability of choosing hospitals and in the probability of perceiving very convenient transportation from 1990–1992 to later years in the 1990s

Using coefficients and the corresponding covariance matrices estimated by the multinomial logit model, we calculated the probability of choosing a specific type of setting as the type of major health facility used for prenatal care [17,19,20]. We further estimated the relative probability of choosing hospitals to choosing non-hospital settings for each time period, and the changes in the relative probability from 1990–1992 to the three later periods in the 1990s [19,20].

The relative probability of choosing large hospitals to choosing non-hospital settings was measured as the ratio of the probability of choosing large hospitals to the probability of choosing non-hospital settings. It is exp(*X*'*b*_1_), where *X *is the set of all explanatory variables and *b*_1 _is the set of coefficients corresponding to the category of visiting medical centres and regional hospitals. Similarly, the relative probability of choosing small hospitals to choosing non-hospital settings is exp(*X*'*b*_2_), where *b*_2 _is the set of coefficients corresponding to local hospitals. The change in the relative probability from 1990–1992 to a specific later period was measured as the ratio of the relative probability for that period to the relative probability for 1990–1992. (The formulas for calculating the 95% confidence interval estimates of these changes are presented in the appendix.)

In the ordered probit model, the values of thresholds (or called "cut points"), together with the values of coefficients, determine the probabilities of falling in various categories of the dependent variable. We used information with respect to coefficients, thresholds and their corresponding covariance matrices to calculate the probability of perceiving very convenient transportation, and estimated the changes in the probability from 1990–1992 to the three later periods in the 1990s [17,20]. The change from 1990–1992 to a specific later period was measured as the probability for that period minus the probability for 1990–1992. (The formulas for calculating the 95% confidence interval estimates of these changes are in the appendix.)

We needed to select a representative case for calculating the probabilities of choosing a specific type of setting as the type of major health facility used for prenatal care and of perceiving very convenient transportation. Referring to the sample characteristics, we chose the following characteristics for such estimation: the child was male and the first child; the mother bore the child before thirty years old, had no abnormal condition during this pregnancy, and was born before 1970, and her ethnicity was Fu-Chien; she had two children, was married, and had senior high school education and an average monthly family income less than 50,000 Taiwanese dollars when she was interviewed in 2000. Based on these characteristics, we calculated the two kinds of aforementioned probabilities for each time period, and for rural and non-rural women, separately.

## Results

### Changes in the relative probability of choosing large hospitals from 1990–1992 to later years in the 1990s

The probability of choosing large hospitals for women in rural areas was significantly higher between 1998 and 1999 than between 1990 and 1992 (Table 4). According to the point estimates, for women in rural areas, the relative probability of choosing large hospitals to choosing non-hospital settings in 1998–1999 was 6.54 times of that in 1990–1992. In contrast, their relative probability of choosing local hospitals in 1998–1999 was only 1.92 times of what it was between 1990 and 1992, and it was not statistically significant.

**Table 4:** The relative probability of choosing hospitals to choosing non-hospital settings as the type of major health facility used for prenatal care

	The probability of choosing a specific type of setting (%)			The relative probability of choosing hospitals to choosing non-hospital settings		The change in the relative probability from 1990–1992			
			
Year	Medical centres or regional hospitals	Local hospitals	Non-hospital settings	Medical centres or regional hospitals	Local hospitals	Medical centres or regional hospitals		Local hospitals	
							
						Point estimate	95%CI	Point estimate	95%CI
Rural areas									
1990–1992	10.6	56.3	33.1	0.32	1.70				
1993–1995	11.8	49.4	38.8	0.30	1.27	0.95	(0.19, 4.73)	0.75	(0.29, 1.95)
1996–1997	14.6	56.1	29.3	0.50	1.92	1.55	(0.38, 6.41)	1.13	(0.47, 2.71)
1998–1999	32.8	51.4	15.8	2.08	3.25	6.54 *	(1.55, 27.62)	1.92	(0.73, 5.05)
									
Non-rural areas									
1990–1992	25.2	30.2	44.6	0.57	0.68				
1993–1995	24.9	31.0	44.0	0.57	0.70	1.00	(0.58, 1.72)	1.04	(0.61, 1.78)
1996–1997	28.6	35.0	36.4	0.79	0.96	1.39	(0.83, 2.33)	1.42	(0.86, 2.34)
1998–1999	25.4	33.2	41.4	0.61	0.80	1.08	(0.62, 1.91)	1.19	(0.70, 2.04)

* p < 0.05.

### Changes in the probability of perceiving transportation to be very convenient from 1990–1992 to later years in the 1990s

Regarding the changes in the probability that women would perceive transportation to be very convenient from the period between 1990 and 1992 to the three later periods in the 1990s, only the change corresponding to 1998–1999 for women in non-rural areas was statistically significant (Table 5). These women were more likely to feel very convenient transportation during the period between 1998 and 1999 than during the period between 1990 and 1992. For a non-rural woman with representative characteristics mentioned previously, she had a 26.8% probability of perceiving that transportation for obtaining prenatal care was very convenient in non-rural areas between 1990 and 1992, and was 8.4% more likely to find it very convenient there between 1998 and 1999. In contrast, women in rural areas did not have this trend, and might be less likely to perceive very convenient transportation between 1998 and 1999 than between 1990 and 1992. According to our point estimates, for a rural woman with aforementioned characteristics, her probability of perceiving transportation as being very convenient was 34.8% between 1990 and 1992, and she was 14.5% less likely to find it very convenient there between 1998 and 1999. This estimate, however, was marginally insignificant, with its upper bound of the 95% confidence interval slightly larger than 0. If our sample size could be a little larger, we would expect such a difference to be statistically significant.

**Table 5:** The probability of perceiving very convenient transportation to and from prenatal care facilities

	Rural areas			Non-rural areas		
		
Year	The probability of perceiving very convenient transportation	Point estimate for the change from 1990–1992	95%CI for the change	The probability of perceiving very convenient transportation	Point estimate for the change from 1990–1992	95%CI for the change
1990–1992	34.8			26.8		
1993–1995	27.8	-7.1	(-24.7, 9.9)	32.6	5.9	(-2.4, 13,7)
1996–1997	27.8	-7.1	(-23.7, 9.4)	32.3	5.5	(-2.1, 12.8)
1998–1999	20.3	-14.5	(-33.0, 0.9)	35.2	8.4 *	(0.01, 15.9)

The numbers are in percentage.

* p < 0.05.

### Other factors related to choice of prenatal care facility

Results from our multivariate analysis revealed there were some other factors related to the choice of prenatal care facility (detailed results available upon request). Women with abnormal health conditions were more likely to seek prenatal health care in large hospitals. Women who bore their babies between age 30 and 34, women who were born before 1970, women with higher education and higher family income were also more likely to seek prenatal care in large hospitals.

## Discussion

In this study of Taiwanese women's choice of prenatal care facility from the early 1990s to the late 1990s, we found that women in rural areas were more likely to go to large hospitals to obtain prenatal care in the late 1990s than in the early 1990s. Moreover, women in rural areas were less likely to perceive that transportation had improved for acquiring prenatal care in the late 1990s than their counterparts in non-rural areas. Our findings suggest that more effort is necessary to help reduce the disparities between women in rural areas and those in non-rural areas.

Since the launch of NHI, recruiting physicians for certain specialties has been challenging for hospitals and medical professional groups, and recruiting for gynaecologic and obstetric departments have had particularly noticeable difficulties. The NHI payment scheme has generally been regarded as a factor associated with such a problem, as gynaecologists and obstetricians complained that the payment level for them is not as good as those for many other specialists [21]. Another factor making gynaecology and obstetrics become unfavourable specialties is that gynaecologists and obstetricians are more likely to be involved in medical disputes than physicians of many other specialties [21]. In the 1990s, there were quite a few cases in which some pregnant women's or lying-in women's family members went to protest or file lawsuits against their gynaecologists and obstetricians because they were not satisfied with the treatments their doctors provided.

Moreover, business has been dropping for gynaecologists and obstetricians as Taiwan's fertility rate has been dropping since the late 1990s. The total fertility rate in 1997 was 1.77, which was about the same level in the early 1990s, but it dropped to 1.56 in 1999, and further to 1.12 in 2005 [22]. The decrease in fertility is significant in both rural and non-rural areas [22]. Business is also uneven, depending on the years considered favourable for giving birth. For example, fertility was much lower in 1998 because it was a Tiger Year, traditionally considered to be unfavourable in Taiwan, and high in 2000, which is a Dragon Year and very favourable. The quickly decreasing and uneven fertility rate also makes gynaecology and obstetrics less attractive specialties.

Regarding the type of health care facility for obtaining prenatal care, we found that women in rural areas were more likely to seek prenatal care in large hospitals in the late 1990s than earlier in the 1990s, a finding that is consistent with results from one study which showed that a large hospital in Taiwan symbolized good quality care in the 1990s [23]. Taiwan's NHI payment scheme has made hospital owners to prefer to use high technology to have their hospitals re-accredited as large hospitals, so that they have had a tendency to compete on a non-price basis [24]. Since the implementation of the NHI, a substantial proportion of small hospitals have gradually exited the hospital market, and a major competition strategy adopted by hospitals is to expand their sizes and increase the use of high technology devices to signal their quality levels [24]. As health care consumers in Taiwan have no reliable information sources regarding the quality levels of health care facilities, some of them may just use the size of a health facility to judge its quality. Particularly, since it is harder for rural residents to collect information on the quality of health care facilities outside their communities, they might be more likely to judge the health care quality of a facility based on its size.

If women in rural areas believe that a larger size of hospital reflects better quality of health care, and choose to leave their living districts to seek prenatal care, it appears reasonable that they would prefer large hospitals rather than smaller health care facilities. The behaviour of enduring inconvenient transportation was also consistent with the finding shown in one research which reported that time cost was a factor with a low elasticity for utilizing ambulatory care in Taiwan in the 1990s [25]. Moreover, the decreases in the numbers of local hospitals and non-hospital health care facilities in rural areas, small cities and towns might also push women in rural areas to be more likely to seek prenatal care in large hospitals. As to why such an effect started to emerge in the late 1990s rather than right after the launch of the NHI, it might be because the influences of the NHI payment scheme and of the dropping fertility on the structure and development of Taiwan's prenatal care market was not so strong in the first a few years of this insurance program, and became stronger a couple of years after the start of the NHI. With respect to such issues, there has been no study reported in the literature, and such research is worthy of more attention.

Given that women in rural areas were more likely to leave their living districts to acquire prenatal care in large hospitals in the late 1990s, it makes sense that women in rural areas were less likely to perceive improved convenience in transportation in the late 1990s than women in non-rural areas. Women in non-rural areas appeared to feel more convenient transportation in the 1990s than earlier in the 1990s. Since the number of health care facilities providing gynaecologic and obstetric care did not increase in the late 1990s, a possible reason for this improvement might be because of the improvements in public transportation systems in non-rural areas. For instance, Taiwan has started developing "mass rapid transit systems" in large cities since the early 1990s. Therefore, it is reasonable that there would be an increased difference in estimation of convenience of transportation to and from prenatal care facility between women in rural areas and their counterparts living in non-rural areas in the late 1990s.

One more point deserving further exploration is whether choosing large hospitals for prenatal care was worth it for women. While our data could not show whether prenatal care services in large hospitals were worse than those offered in other settings, such as clinics and public health stations, some previous studies indicated that women seeking prenatal care in clinics were more likely to obtain adequate prenatal care than those seeking such care in hospitals one year after the NHI program started [6,10]. Our study did not focus on "measures for adequacy of prenatal care." It would require further study to investigate the consequences of rural women's actions of leaving their local communities and going to large hospitals for prenatal care in the post-NHI period in terms of the quality of prenatal care received and the corresponding birth outcomes.

This study, which examined disparities between rural and non-rural areas, offers new information regarding the type of major health care facility used, and women's perception of the convenience of transportation for obtaining prenatal care. It discusses in detail the links of the trend in Taiwanese women's choice of prenatal care facility with developments in Taiwan's NHI, health care market, fertility and public transportation. It thus adds important information to the field of prenatal care. Our findings should be of particular use to Taiwan and countries with similar concerns and conditions for their future reforms in the delivery of prenatal care.

How to improve access to prenatal care in rural areas should be an important challenge. The literature has indicated that women who seek obstetrical care outside their local communities are more likely to have complicated deliveries, larger chances of prematurity, and more need for neonatal care for their children [26]. Previous research has also suggested that fewer pregnancy-related health services resource in rural areas adversely affects utilization of such health care in these areas [27]. Rural women are unavoidably facing higher risks for birth delivery, since they usually have to leave their community to deliver their children. Such a circumstance makes "use of good quality prenatal care" even more essential for women in rural areas. While it does not seem efficient to increase the number of health care facilities offering prenatal care in rural areas, some other strategies should be seriously considered and tested. For instance, it should be helpful to improve the quality of prenatal care offered in rural public health stations by recruiting good gynaecologists and obstetricians to provide services in the health stations on a periodic and regular basis, and to construct a good referral system for prenatal care using public health stations as the base. It should also be helpful to provide rural women with more convenient transportation services to and from prenatal health care facilities. Furthermore, it should be helpful to disseminate health information on the importance of and the criteria for adequate prenatal care to help pregnant women make better choices regarding prenatal care.

This study focused on reporting results based on data for 1,575 children in the 4,820 available cases, as previously mentioned. The reason is that we concerned correlation among multiple cases from a same woman, since the two statistical models we used, the multinomial logit model and the ordered probit model, cannot not handle this kind of correlation problem. However, for the purpose of sensitivity analysis, we have also done analysis based on the 4,820 cases (results not shown). While more coefficients were statistically significant in results from analysing the 4,820 cases, these results do not conflict with conclusions we made with regard to comparison between rural and non-rural areas according to our results from analysing the 1,575 cases.

There are two main limitations for this study. The first pertains to recall bias, a problem inherent in most retrospective studies that make of surveys. Nonetheless, we believe that such recall bias in our study should not be serious. It has been shown that maternal recall of births five to seven years earlier is highly accurate, and maternal reports of prenatal events more than a decade after the birth are still reasonably accurate, especially events they directly participated in or information they have been told [28-31]. The literature has also indicated that health care users can reliably report factual information such as the travelling time for obtaining ambulatory care [32]. Since most Taiwanese women only have one or two children and the recall period for this study is no more than ten years, it should not be hard for them to remember which health care facilities they used for prenatal care, and how they felt about the transportation to and from the facilities.

The second limitation is that our analysis only compared different cohorts of women giving births in the 1990s without controlling for some unobserved characteristics, such as attitudes and beliefs with regard to childbearing, prenatal care, and health care quality differences among different types of facilities. We were not able to further discuss how such characteristics that might vary among different cohorts of women might interact with the NHI influences on the prenatal care market, and subsequently result in changes in women's choice of prenatal care facilities. If we could also compare the experiences of prenatal care utilization for a specific cohort before and after the implementation of the NHI, we would be able to furnish more knowledge in this area.

## Conclusion

In conclusion, we found that the women in rural areas were more likely to seek prenatal care in large hospitals, but were not more likely to perceive very convenient transportation to and from prenatal care facilities in the late 1990s than in the early 1990s. In contrast, women in non-rural areas did not have a stronger tendency to seek prenatal care in large hospitals in the late 1990s than in earlier periods. In addition, they did perceive an improvement in transportation for acquiring prenatal care in the late 1990s. More efforts should be undertaken to reduce these disparities and improve access to prenatal care of good quality in rural areas.

## Competing interests

The author(s) declare that they have no competing interests.

## Authors' contributions

Likwang Chen designed the study, led the data collection and statistical analysis, and drafted the manuscript. Chi-Liang Chen participated in designing the framework of statistical analysis and preparing the manuscript. Wei-Chih Yang participated in analyzing data analysis and preparing the manuscript. All authors read and approved the final manuscript.

## Appendix

Formulas for calculating the 95% confidence interval estimates of changes corresponding to the three later periods in the 1990s

Changes in the Relative Probability of Choosing Hospitals from 1990–1992 to Later Years in the 1990s

The 95% confidence interval estimates of changes corresponding to the three later periods in the 1990s for non-rural areas were calculated as follows:

$$\begin{array}{l} \text{For }1993-1995:\exp[b_{1993\_1995}\pm z_{1-0.05/2}\times \hat{s}e(b_{1993\_1995})]\\ \text{For }1996-1997:\exp[b_{1996\_1997}\pm z_{1-0.05/2}\times \hat{s}e(b_{1996\_1997})]\\ \text{For }1998-1999:\exp[b_{1998\_1999}\pm z_{1-0.05/2}\times \hat{s}e(b_{1998\_1999})] \end{array}$$

For rural areas, the 95% confidence interval estimates for the three periods were respectively calculated as follows:

$$\begin{array}{l} \exp[(b_{1993\_1995}+b_{Rural*1993\_1995})\pm z_{1-0.05/2}\times {\left((\hat{\mathrm{var}}(b_{1993\_1995})+\hat{\mathrm{var}}(b_{Rural*1993\_1995})+2\hat{\mathrm{cov}}(b_{1993\_1995},b_{Rural*1993\_1995})\right)}^{1/2}]\\ \exp[(b_{1996\_1997}+b_{Rural*1996\_1997})\pm z_{1-0.05/2}\times {\left((\hat{\mathrm{var}}(b_{1996\_1997})+\hat{\mathrm{var}}(b_{Rural*1996\_1997})+2\hat{\mathrm{cov}}(b_{1996\_1997},b_{Rural*1996\_1997})\right)}^{1/2}]\\ \exp[(b_{1998\_1999}+b_{Rural*1998\_1999})\pm z_{1-0.05/2}\times {\left((\hat{\mathrm{var}}(b_{1998\_1999})+\hat{\mathrm{var}}(b_{Rural*1998\_1999})+2\bar{\mathrm{cov}}(b_{1998\_1999},b_{Rural*1998\_1999})\right)}^{1/2}] \end{array}$$

Changes in the Probability of Perceiving Very Convenient Transportation from 1990–1992 to Later Years in the 1990s

In our model, there were three thresholds: *K*_1_, *K*_2_, *K*_3_. The 95% confidence interval estimates of changes in the probability of perceiving very convenient transportation corresponding to the three later periods for non-rural areas were respectively calculated as follows:

$$\begin{array}{l} \text{For }1993-1995:\varphi (K_{3}-X^{\prime }b)*[b_{1993\_1995}\pm z_{1-0.05/2}\times \hat{s}e(b_{1993\_1995})]\\ \text{For }1996-1997:\varphi (K_{3}-X^{\prime }b)*[b_{1996\_1997}\pm z_{1-0.05/2}\times \hat{s}e(b_{1996\_1997})]\\ \text{For }1998-1999:\varphi (K_{3}-X^{\prime }b)*[b_{1998\_1999}\pm z_{1-0.05/2}\times \hat{s}e(b_{1998\_1999})] \end{array}$$

*φ *is the density function of the standard normal distribution.

For rural areas, the three interval estimates were:

$$\begin{array}{l} \varphi (K_{3}-X^{\prime }b)*[(b_{1993\_1995}+b_{Rural*1993\_1995})\pm z_{1-0.05/2}\times {\left((\hat{\mathrm{var}}(b_{1993\_1995})+\hat{\mathrm{var}}(b_{Rural*1993\_1995})+2\hat{\mathrm{cov}}(b_{1993\_1995},b_{Rural*1993\_1995})\right)}^{1/2}]\\ \varphi (K_{3}-X^{\prime }b)*[(b_{1996\_1997}+b_{Rural*1996\_1997})\pm z_{1-0.05/2}\times {\left((\hat{\mathrm{var}}(b_{1996\_1997})+\hat{\mathrm{var}}(b_{Rural*1996\_1997})+2\hat{\mathrm{cov}}(b_{1996\_1997},b_{Rural*1996\_1997})\right)}^{1/2}]\\ \varphi (K_{3}-X^{\prime }b)*[(b_{1998\_1999}+b_{Rural*1998\_1999})\pm z_{1-0.05/2}\times {\left((\hat{\mathrm{var}}(b_{1998\_1999})+\hat{\mathrm{var}}(b_{Rural*1998\_1999})+2\hat{\mathrm{cov}}(b_{1998\_1999},b_{Rural*1998\_1999})\right)}^{1/2}] \end{array}$$

## Pre-publication history

The pre-publication history for this paper can be accessed here:


